# Case Report: Hemidiaphragm Paralysis Results in Reduced Blood Oxygen Saturation, Increased Respiratory Rate, and Severe Dyspnea in Supine and Prone Positions due to Impaired Abdominal Breathing

**DOI:** 10.3390/life16040634

**Published:** 2026-04-09

**Authors:** Akos Koller, Szonja Decker, Johanna Takács, Andrea Harangozo, Bela Faludi, Tamás Horváth

**Affiliations:** 1 Research Center for Sports Physiology, Hungarian University of Sports Science, 1123 Budapest, Hungary; decker.szonja@mayo.edu (S.D.); horvath2.tamas@tf.hu (T.H.); 2 Department of Morphology & Physiology, Faculty of Health Sciences, Semmelweis University, 1085 Budapest, Hungary; 3 HUN-REN-SE, Cerebrovascular and Neurocognitive Disorders Research Group, Institute of Clinical Pathophysiology, Semmelweis University, 1085 Budapest, Hungary; 4 Department of Cell and Molecular Physiology, New York Medical College, Valhalla, NY 10595, USA; 5 Mayo Clinic Hospital, Rochester, MI 55905, USA; 6 Department of Social Sciences, Faculty of Health Sciences, Semmelweis University, 1085 Budapest, Hungary; spss.stat@gmail.com; 7 Pulmonary and Intensive Care Specialists of New Jersey, East Brunswick, NJ 08816, USA; amharangozo@gmail.com; 8 Department of Neurology, University of Pécs, 7623 Pécs, Hungary; faludi.bela@pte.hu

**Keywords:** hemidiaphragm paralysis, breathing mechanics, breathing efficiency, body positions

## Abstract

**Background:** The breathing cycle consists of abdominal breathing (AB), for which the diaphragm is responsible, and thoracic breathing (TB), generated by the intercostal muscles. Contraction of the two portions of the diaphragm accounts for 80% percent of inspiration. While bilateral diaphragmatic paralysis causes severe shortness of breath, hemidiaphragm paralysis (HDP) gives fewer symptoms at rest, making it difficult to recognize and diagnose. Because this condition is rare, little is known regarding its consequences on breathing efficiency. **Hypothesis:** Based on previous studies, we hypothesized that body positions substantially affect the efficiency of breathing in a patient with unilateral hemidiaphragm paralysis and the corresponding physiological parameters. **Aims:** To measure and compare the amplitudes of abdominal and chest movements in different body positions in an individual with HDP and measure parameters indicating breathing efficiency. **Patient and Methods:** The patient had HDP due to iatrogenic phrenic nerve injury. Changes in the circumference of the abdomen and chest were measured during inhalation and exhalation with respiratory plethysmography belts (placed on standardized reproducible positions on the chest and abdomen) in different body positions: sitting (SI), standing (ST), lying (SU) and prone (PR). Breathing frequency was calculated, and blood oxygen saturation (SpO_2_) was measured with a pulse oximeter. **Results**: The percentage (%) contributions of abdominal breathing were SI: 16.0; ST: 50.3; SU: −53.5; PR: 1.1. A negative sign shows paradoxical breathing. Blood oxygen saturation (SpO_2_) in the four positions was SI: 93%; ST: 93%; SU: 82%; and PR: 82%, whereas the respiratory rate (1/min) was SI:19.4; ST: 15.0; SU: 37.5; PR: 35.9. **Conclusions:** Body position markedly influences the relative contributions of abdominal and thoracic breathing and overall respiratory efficiency in patients with hemidiaphragm paralysis; abdominal breathing in the supine and prone positions is greatly reduced leading to decreased blood oxygen saturation, a compensatory increase in respiratory rate, and severe dyspnea even at rest.

## 1. Introduction

The movement of the diaphragm primarily drives abdominal breathing, whereas thoracic breathing involves additional accessory respiratory muscles in the upper thoracic cage [1,2,3,4,5]. The diaphragm plays a pivotal role in both abdominal and thoracic breathing (AB and TB) by coordinating movements that influence both cavities, causing the chest and abdomen to move in synchrony during tidal breathing as its contraction expands both structures [2,6]. Normal quiet breathing involves both abdominal and thoracic breathing, in which the diaphragm is responsible for approximately 70% of the inhaled air volume during inspiration [7,8,9,10,11,12]. Even though these patterns can be separated, normal, quiet breathing involves both breathing patterns [1,2].

### Hemidiaphragm Paralysis (HDP)

The diaphragm, the primary muscle for inspiration, consists of the right and left hemidiaphragms, which are inserted into a central tendon but can function independently. Each hemidiaphragm is innervated separately by the right and left phrenic nerves, originating from motoneurons in the C3–C5 spinal cord region [13,14]. Typically, the right hemidiaphragm sits 1.5 to 2.5 cm higher than the left [14]. During contraction, the diaphragm generates negative intrathoracic pressure, allowing the lungs to inflate. This contraction causes the diaphragm to descend, expanding the thoracic cavity vertically and exerting force on its attachment to the rib cage. The resulting decrease in pleural pressure and increase in abdominal pressure raises trans-diaphragmatic pressure [3,13,15,16,17,18]. The crucial role of the diaphragm becomes evident when the phrenic nerve is damaged, typically affecting one side (unilateral) rather than both sides (bilateral) [19]. The most frequent causes of phrenic nerve injury include trauma, such as thoracic or cardiac surgery [19,20].

Other potential causes include nerve compression, exposure to ice slush during cardiac surgery, certain medications, demyelinating disorders (neuropathy, such as inflammatory demyelinating polyneuropathy: CIDP), cervical spinal cord injury, and congenital conditions [21]. When the phrenic nerve is injured on one side, the affected hemidiaphragm loses its ability to contract. At rest, this impairment may go unnoticed, but it can reduce respiratory function, leading to exertional dyspnea, a common symptom in both unilateral and bilateral diaphragm paralysis [22]. Previous studies using ultrasound imaging revealed that HDP prevents the lung from achieving proper expansion [23] and diminishes ventilation due to asynchrony of lungs in different body positions in unilateral diaphragm paralysis [24], suggesting a change in the contribution of abdominal and thoracic breathings.

Yet the mechanical contribution and/or impairment of abdominal and thoracic breathing mechanics has not been studied. Based on our previous studies [1], we hypothesized that abdominal breathing, indicating diaphragm function, would be severely reduced in supine and prone positions, resulting in reduced blood oxygen saturation and increased respiratory rate.

## 2. Materials and Methods

### 2.1. Study Participant

**Figure 1:**
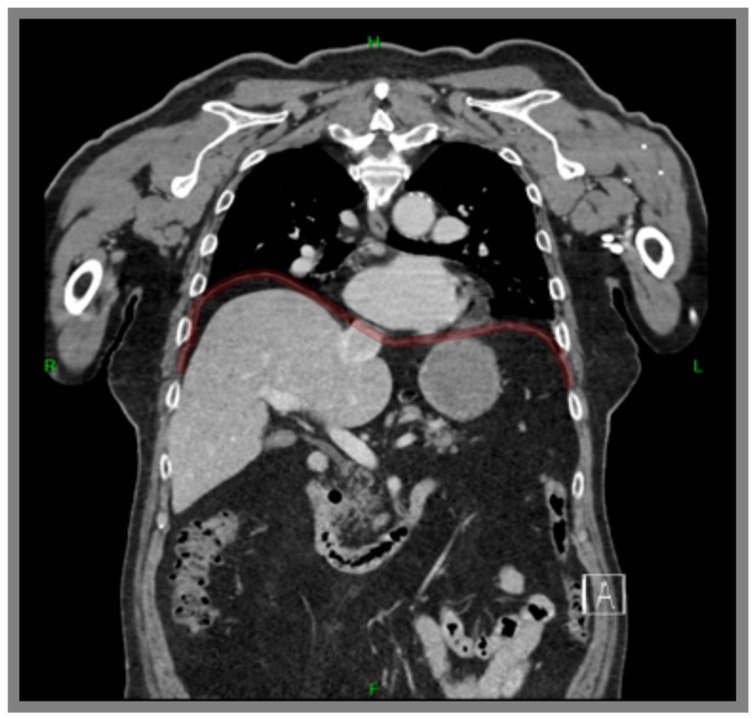
Computer tomography (CT) image of the studied patient with right hemidiaphragm paralysis (the contour is highlighted by a red line). On the right side, the paralyzed hemidiaphragm is substantially elevated in the thorax, resulting in spatial limitation for the lung to expand and relocation of the liver and the heart.

The general characteristics of the patient with hemidiaphragm paralysis (HDP) (Figure 1) are included in Table 1. The participant received both verbal and written explanations about the procedures and provided informed consent by signing a consent form. The research was approved by the Hungarian University of Sports Sciences Research Ethics Committee (approval number TE-KEB/13/2023).

### 2.2. Measurements and Calibration of Respiratory Belts

The movements/expansions of the abdomen and thorax were measured with respiratory belts (ADInstruments, TN1132/ST. Unit B, Bishops Mews, Transport Way, Oxford OX4 6HD, UK). The belts were connected to the ADInstruments Power Lab 4/30 data acquisition unit. The respiratory belts were positioned at the level of the tenth thoracic vertebra and the fourth lumbar vertebra as described earlier [1,4,25,26,27,28,29,30], and the maximum and minimum between 0 and 100 mV excursion were adjusted to set the resting oscillation around 50 mV. The ambient temperature of the examination room was maintained at 21 degrees Celsius with normal humidity.

Blood pressure and heart rate were measured with an Omron Hem-907 (Omron Healthcare Co., Ltd., Kyoto, Japan) monitor. Blood oxygen saturation (SpO_2_) was measured by pulse oximetry (Beurer PO45, Beurer GmbH, Ulm, Germany) placed on the index finger. Body mass, body height and body mass index (BMI) were also measured [31].

### 2.3. Four Body Positions

**Figure 2:**
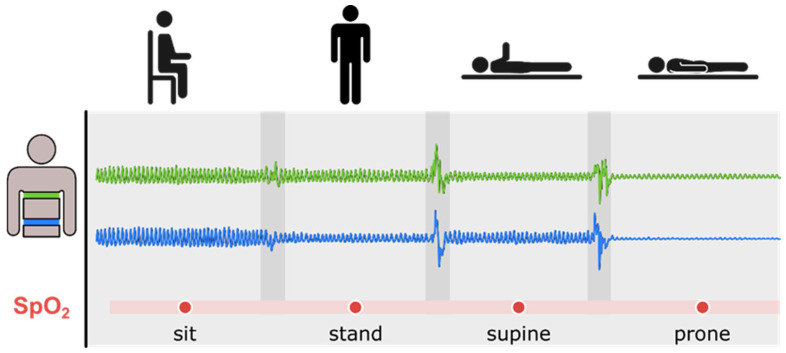
Experimental protocol: schematic diagram of the four body positions as well as the corresponding respiratory belt signals (green: thoracic, blue: abdominal). For clarity in the figure, one arm is lifted to indicate the supine position as opposed to the prone position. The thoracic belt and the abdominal belt were aligned with the vertebral positions (Th10 and L4). Blood oxygen saturation (SpO_2_) and respiratory rate were also measured in each position. Five measurements were performed in each position, and the mean ± SEM was calculated.

Changes in both abdominal and thoracic movements/expansions were measured in four different body positions (Figure 2). Between each body position, a one-minute rest period was kept, with each measurement lasting ~25 min.

1. Sitting position (SI): The participant’s feet were placed flat on the ground, with a 90-degree angle between the thighs and lower legs. The spine did not touch the back of the chair, as back support can influence breathing patterns [32].

2. Standing position (ST): The participant was in the standard standing position [33].

3. Supine position (SU): The limbs of the participant were extended, feet close together, arms alongside the body, and the face was directed toward the ceiling.

4. Prone position (PR): The participant was allowed to turn his head to the side for comfort.

### 2.4. Data Recording and Signal Processing

Thoracic and abdominal breathing signals were recorded and analyzed using an ADInstruments PowerLab 4/30 data logger and ADInstruments LabChart software (version 8) as described earlier [1].

## 3. Results

**Figure 3:**
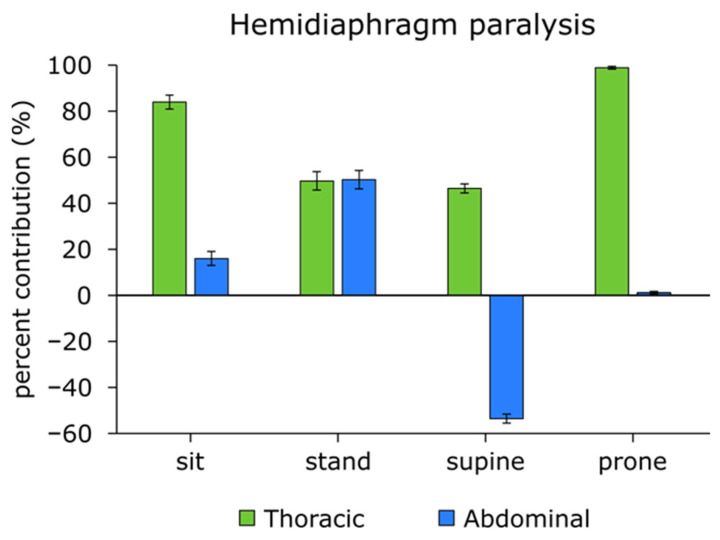
Percentage contribution of abdominal and thoracic movements to the total respiratory cycle in a patient with hemidiaphragm paralysis (mean ± SEM *n* = 5). To indicate paradoxical breathing, the abdominal contribution in the supine position is shown as a negative percentage.

In the hemidiaphragm paralyzed patient, the ratio of abdominal–thoracic breathing to the total respiratory cycle changed across different body positions. As shown in Figure 3, the percentage contribution of abdominal breathing in each position was: SI: 16.0%, ST: 50.3%, SU: −53.5%, PR: 1.1%. The highest level of abdominal breathing was observed in the sitting position (84%), while paradoxical abdominal movement (abdomen moved inside during inhalation) occurred in the supine position, as indicated by the negative value (−53.5%).

**Figure 4:**
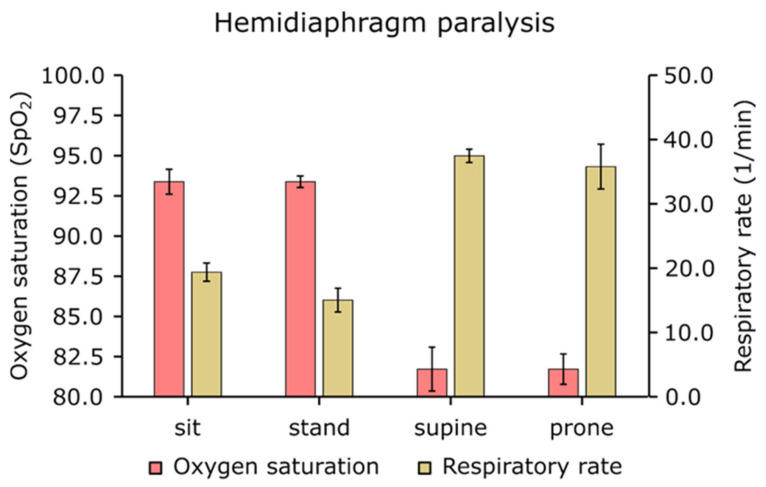
Oxygen saturation and respiratory rates in different body positions in a patient with hemidiaphragm paralysis (mean ± SEM *n* = 5). The highest respiratory rate was measured at the supine position when the oxygen saturation was the lowest.

Paradoxical breathing (the abdomen and chest move in opposite directions) was present in the supine position, resulting in inefficient, shallow inhalation and exhalation patterns, which is an energy-consuming and suboptimal condition. In this position, the SpO_2_ significantly dropped (SI: 93% vs. SU: 82%, PR: 82%), and as a consequence, the respiratory frequency increased (Figure 4).

The values of blood oxygen saturation (SpO_2_) were position dependent: SI: 93%, ST: 93%, SU: 82%, and PR: 82%. Correspondingly, the respiratory rate (RR, breaths per minute) in the respective positions was SI: 19.4, ST: 15.0, SU: 37.5, PR: 35.9. The lowest oxygen saturation levels occurred in the supine position, where respiratory rate was the highest, and abdominal movement was paradoxical (Figure 4).

## 4. Discussion

The salient findings of the present study are that in a patient with hemidiaphragm paralysis, body position markedly influences the relative contributions of abdominal and thoracic breathing and overall respiratory efficiency. Reduced abdominal breathing in the supine and prone positions leads to decreased oxygen saturation and a compensatory increase in respiratory rate, resulting in severe dyspnea even at rest. In the future, larger studies are needed to further evaluate the effect of body position on different breathing patterns in hemidiaphragm paralysis.

A previous study showed that body position changes the abdominal and thoracic breathing movements between healthy individuals and competitive athletes [1]. The current study further strengthens this finding by showing that in the case of hemidiaphragm paralysis, abdominal and thoracic breathing movements change in different positions. Hemidiaphragm paralysis is a serious health condition that is often overlooked [34]. Several studies indicate that unilateral diaphragm paralysis can impact sleep quality and contribute to sleep apnea [34,35,36]. Additionally, exertional dyspnea is a common symptom, reducing a patient’s ability to exercise [22]. The combined inability to sleep or engage in physical activity can significantly affect an individual’s quality of life.

Hemidiaphragm paralysis frequently goes unnoticed because it often occurs post-surgery and can be masked by underlying health issues [19,20]. Consequently, symptoms may not be immediately recognized as a new condition. Following phrenic nerve denervation, a ∼40% decrease in Pdi amplitude during airway occlusion was observed immediately, but this reduction improved to ∼20% after 14 days. Over time, compensatory activation of inspiratory-related muscles other than the diaphragm may contribute to the increased Pdi during higher-force behaviors [37], with accessory muscles progressively compensating as diaphragm function declines.

Compared to rodent studies, it is an important consideration that their natural state and the position in which measurements are taken are horizontal [37]. In contrast, in humans, the natural state is upright, and measurements are typically performed in sitting positions. Previous studies have found no significant differences in blood gas levels between healthy individuals and those with hemidiaphragm paralysis during resting breathing [18]. However, when humans are assessed in a horizontal position, significant changes in ventilatory capacity and dead space may occur, resulting in reduced blood gas levels and increased respiratory rate, as shown in the present study (Figure 3 and Figure 4). Thus, body position plays a crucial role in revealing functional impairments associated with hemidiaphragm paralysis. Previous studies have assessed hemidiaphragm and diaphragm paralysis using various clinical measurements like diaphragm fluoroscopy, fluoroscopic sniff test, ultrasonography, computed tomography, and Pdi(max) measurements [38], while magnetic phrenic nerve stimulation is currently the most precise technique for diagnosing phrenic nerve paralysis. Nevertheless, all these assessments are conducted in a clinical setting. It has been previously stated that the effect of the diaphragm on tidal breathing is most apparent in the supine posture [39]. Our findings align with previous research indicating that abdominal breathing contributes most in the supine position [38,40].

The present study revealed that, in a hemidiaphragm paralytic patient, in the supine position, a pronounced paradoxical abdominal motion and an abnormal thoracic movement pattern are present (they work in opposite directions), resulting in a lower blood oxygen saturation level and increased respiratory rate (Figure 3 and Figure 4). Similarly, in the prone position, abdominal breathing is absent. In sitting and standing positions, thoracic breathing compensates for the lack of abdominal (diaphragm) breathing, which, however requires increased energy consumption for respiration, resulting in dyspnea even at rest.

## 5. Clinical Consequences

Previously, it has been recognized that right-sided phrenic nerve injury after shoulder surgery can lead to serious dyspnea in a patient, due to hemiparesis of the diaphragm [39], which is difficult to recognize or diagnose, especially in emergencies, unless X-ray or ultrasounds are available. Use of respiratory belts is affordable and diagnostically helpful. Other studies have shown that diaphragm muscle atrophy (either due to reduced diaphragm muscle mass or neural dysfunction) contributes to low physical capacity in COVID-19 survivors [41].

An interesting study found an exercise-induced diaphragm fatigue in an athlete with a spinal cord injury and suggested that, in addition to high levels of exercise hyperpnea, factors other than ventilation, such as posture, are responsible for the fatigue [42].

In general, in hemidiaphragm paralysis, patients may be asymptomatic in a resting condition and become apparent during physical activity, especially if it is accompanied by obesity [43]. Others also reported compensatory effects following unilateral diaphragm paralysis, observing that over time, breathing gets easier, and less of a paradoxical movement is seen with inspiration, allowing for better gas exchange [37]. In most cases, patients will not require treatment; however, they may need CPAP help during supine positions while sleeping or lying in bed.

Recognition of such conditions, especially after open chest surgery, is important as the patient may require the use of continuous positive airway pressure (BiPAP or BiPAP S or frequency controlling BiPAP ST-machines) for sleeping in supine and back positions. In addition, such patients have difficulties with—among others—ECG, UH, CT, and MRI diagnostic tests because they are routinely performed in a supine position. Another less-considered and unrecognized difficulty is that such a patient cannot breathe if submerged in water because the chest cannot move against the side-pressure of water, thus the patient cannot generate negative intrathoracic pressure and cannot breathe, even though the mouth is out of the water and the airways are clear, leading to suffocation.

## 6. Conclusions

To our knowledge, this is the first study to specifically evaluate breathing mechanics in hemidiaphragm paralysis using respiratory belts across multiple body positions. We suggest that this technique may serve as a valuable, non-invasive, and efficient screening tool for diagnosing this condition.

The present findings underscore the importance of evaluating respiratory function across different body positions, as the relative contributions of abdominal and thoracic breathing are strongly influenced by posture and diaphragmatic performance. In the supine and prone positions, abdominal breathing and its effectiveness are significantly reduced in patients with hemidiaphragm paralysis. This impairment leads to decreased oxygen saturation (SpO_2_), an increased respiratory rate, and a higher energy cost of breathing, ultimately resulting in severe dyspnea even at rest.

## Figures and Tables

**Table 1:** General characteristics of the patient.

Body weight	84 kg
Height	170 cm
Body Mass Index (BMI)	29 kg/m^2^
Blood pressure	134/90 mmHg
Heart Rate	73/min
Medications:	
Type 2 diabetes: Jardiance (1 × 10 mg/day)	
Hypertension: Coverex-AS (1 × 10/2.5 mg/day), Propafenone 2 × 150 mg/day, Eliquis (2 × 5 mg/day)	

## Data Availability

Data can be obtained from the corresponding author upon request.
